# Results of liver transplantation with donors older than 80 years: a case control study 

**Published:** 2017

**Authors:** Francisco Javier León Díaz, José Luis Fernández Aguilar, Belinda Sánchez Pérez, Custodia Montiel Casado, José Manuel Aranda Narváez, José Antonio Pérez Daga, Miguel Ángel Suárez Muñoz, Julio Santoyo Santoyo

**Affiliations:** *Liver Transplant Unit, Regional Hospital, Málaga, Spain*

**Keywords:** Liver transplantation, Older donors, Octogenarians

## Abstract

**Aim:**

The inclusion of elderly donors can increase the pool of organs available for transplant.

**Background:**

To compare clinical outcomes and survival rates in patients who received livers from donors aged ≥ 80 years vs. younger donors.

**Methods:**

We considered all liver transplantations performed in our unit between January 2006 and January 2015. Twelve patients received liver from a cadaveric donor aged ≥ 80 years (study group) and their outcomes were compared with those of patients who received liver from a younger donor (control group). This study was carried out to analyze the characteristics of donors and recipients, as well as the clinical course and survival of recipients.

**Results:**

Statistically significant differences were observed in donors' age (55.6 ± 14.4 vs. 82.7 ± 2.7 years, p < 0.001), donors' ICU stay (p = 0.008), donors' ALT levels (p = 0.009) and donors' AST levels (p = 0.01). Statistically significant differences were found in ischemia time (p < 0.05). In total, 8.3% of the recipients of liver from a donor aged < 80 required retransplantation vs. 25% of recipients of donor’s ≥ 80 years. Patient survival at one, three and five years was 89%, 78.6% and 74.5%, respectively vs. 83.4%, 79.4% and 59.6% for the study group.

**Conclusion:**

Livers from older donors can be safely used for transplantation with acceptable patient survival rates. However, graft survival rates are lower for recipients of livers from older donors as compared to younger donors, and survival only increased with retransplantation.

## Introduction

 Mortality in candidates waiting for liver transplants increases by 10% per year in Spain. This is due to the large number of candidates on the waiting list for an orthotopic liver transplantation and the limited number of liver donors (1). Thus, in order to expand the pool of donors, the selection criteria were broadened to include older donors, although there is no general consensus on the safety of this practice (2). On the one hand, some studies associate the use of organs from older donors with higher rates of dysfunction and primary graft failure (3,4). On the other hand, other studies confirm the safety and optimal outcomes of transplants from older donors if patients are appropriately selected (5,6). The objective of this study was to compare the clinical outcomes and survival rates of patients who received a liver from a donor aged ≥ 80 years vs. younger donors. 

## Methods

We considered all liver trasplantations performed in our unit between January 2006 and January 2015 and identified a total of 12 cadaveric donors aged ≥ 80 years. A retrospective case – control study design was selected using a 1:2 ratio. Donor and recipient variables were matched to a control group of 24 patients, who were transplanted from younger donors immediately before or after each index case. During the procurement phase, liver biopsies were obtained at the discretion of the surgeon. Exclusion criteria were the presence of steatosis ≥30%, bridging fibrosis or hepatitis. Post-transplant biopsy was considered positive for steatosis if ≥30%. We analyzed both donor characteristics (including age, sex, body mass index [BMI], aspartate aminotransferase [AST] / alanine aminotransferase [ALT], bilirubin, presence of steatosis and ischemia time) and recipient variables (including age, sex, BMI, etiology of liver disease, Model for End-Stage Liver Disease score [MELD], time on the waiting list, liver function parameters, pre- and post- transplant ICU stay, hospital stay, presence of primary graft non-function [PGNF], initial poor graft dysfunction [IPGD], need for retransplantation, reoperation, rejection, infection, vascular and biliary complications, hospital re-stay and graft survival). IPGD was defined as the presence of one or more of the following previously defined postoperative laboratory results suggestive of liver injury and dysfunction: bilirubin >10mg/dL on day 7, international normalized ratio >1.6 on day 7, and alanine or aspartate aminotransferases >2000 IU/L within the first 7 days 7.

All patients who were selected for liver transplantation for HCC met the Milan criteria, i.e. a single tumour less than or equal to 5 cm in diameter or no more than three nodules not exceeding 3 cm.

**Table 1:** Donor characteristics

	DONORS < 80 y	DONORS ≥ 80 y	p
Numbers	24	12	
Age (y)	55.6 ± 14.4	82.7 ± 2.7	0.001
ICU stay (d)	2.33 ± 2.04	1.58 ± 0.7	0.008
AST (U/L)	64.1 ± 75.7	32.17 ±12.5	0.01
ALT (U/L)	54.6 ± 67.8	21.33 ± 9.6	0.009
Bi (mg/dl)	0.75 ± 0.83	0.73 ± 0.26	0.17
Steatosis	3 (12.5%)	0 (0%)	0.2

Intensive care unit (ICU); aspartate aminotransferase (AST); Alanine aminotransferase (ALT); bilirubin (Bi).

**Table 2:** Recipient characteristics

	DONORS < 80 y	DONORS ≥ 80 y	p
Numbers	24	12	
Age (y)	56.3 ± 10.14	59.5 ± 4.8	0.09
MELD	16 ± 9.1	12.9 ± 4.3	0.07
Time in list if OLT (d)	184.3 ± 220.1	181.8 ± 148.2	0.38
Etiology of liver diseases			
Alcohol	8 (33.3%)	8 (72.7%)	0.03
Hepatitis C virus	3 (12.5%)	0 (0%)	0.07
CHC	5 (20.8%)	3 (27.3%)	0.08
Others	8 (33.3%)	1 (9.1%)	0.06

Model for End-Stage Liver Disease score (MELD); OLT, orthotopic liver transplantation.

**Figure 1:**
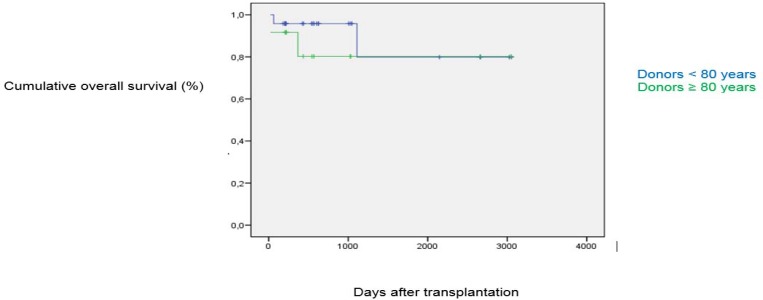
Patient survival.

**Figure 2:**
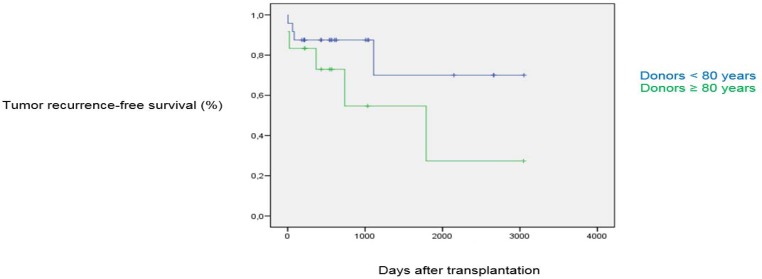
Graft survival

**Statistical Analysis**

Differences between mean values were evaluated using Student's t-test or the Mann-Whitney U test. Differences in categorical variables between the two groups were evaluated using the chi-square test. Postoperative graft survival was computed from the day of OLT to the last follow-up visit or death or retransplantation. Survival rates were estimated by the life table method with differences compared using the log-rank test. Data were processed using SPSS 15.0 software (SPSS Inc, Chigaco, Ill, USA). A P value ≤0.05 was considered statistically significant. 

## Results

Donor characteristics, biochemical parameters and ischemia time are shown in Table 1. Obviously, significant differences were found in the age of donors between the study group and the control group (55.6 ± 14.4 vs. 82.7 ± 2.7 years, p < 0.001). No statistically significant differences were observed in sex, biochemical parameters (except for ALT levels p = 0.009 and AST levels p = 0.01) or steatosis. There were significant differences in donors' ICU stay (2.3 ± 2.01 vs. 1.58 ± 0.67 for the study group, p = 0.008). Statistically significant differences were found in ischemia time: total ischemia (418.6 ± 132.01 of donors < 80 vs. 405.08 ± 67.2), warm ischemia (53.25 ± 19.02 vs. 58.42 ± 10.62 of the study group) and cold ischemia (365.3 ± 123.53 vs. 346.67 ± 69.34 of donor’s ≥80). 

Recipient characteristics and postoperative data are shown in Tables 2 and 3. The age of recipients was similar in both groups, as well as time on the waiting list, MELD and type of transplant. Of note is that the prevalent etiologies of liver disease in the control group were liver cancer, hepatitis C virus and alcohol-abuse, whereas the prevalent causes of liver disease in the control study were liver cancer and alcohol-abuse. There were no statistically significant differences concerning indication of transplantation for hepatitis C virus (18.8% for the control group vs. 0% for the study group, p = 0.05). 

No statistically significant differences were found concerning the type of postoperative complication. In total, 8.3% of the recipients of donors < 80 years required retransplantation vs. 25% of recipients of donors ≥ 80 years. The median follow-up time was 22 months (range: 6 - 108). Patient survival (Figure 1) for the control group at one, three and five years was 95.8%, 79.9% and 79.9%, respectively, vs. 91.7%, 80.2% and 80.2% for the study group. Graft survival (Figure 2) for the control group at one, three and five years was 87.5%, 87.5% and 70%, respectively, vs. 83.3%, 54.7% and 27.3% for the study group, indicating no statistically significant differences.

## Discussion

Despite evidence that use of organs from older donors is associated with liver dysfunction and lower survival rates, the available evidence is not conclusive. If older donors are appropriately selected by eliminating extra risk factors, there is no strong evidence to discourage the use of grafts from older donors (8,9). According to our experience, there were no statistically significant differences between recipients of older donors and those of younger donors. 

The results obtained show that the incidence of primary graft non-function was similar in both groups. However, initial poor graft dysfunction (45.8% for the control group vs. 16.7% for the study group) was higher in the control group, although the differences were not statistically significant. 

Some studies report higher rates of arterial complications in recipients of older donors (10). The incidence of arterial and biliary complications doubles for grafts from donors aged ≥ 80 years, although the differences were not statistically significant. In total, 8.3% of the recipients of a liver from a donor aged < 80 years required retransplantation vs. 28% of recipients of donors ≥ 80 years.

**Table 3:** Postoperative data

	DONORS < 80 y	DONORS ≥ 80 y	p
Numbers	24	12	
Cold ischemia time (min)	365.3 ± 123.5	346.7 ± 69.3	0.04
Warm ischemia time (min)	53.25 ± 19	58.4 ± 10.6	0.03
Total ischemia time (min)	418.6 ± 132	405.1 ± 67.2	0.02
ICU stay (d)	4.6 ± 3.5	5.7 ± 6.3	0.14
Hospital stay (d)	17.25 ± 12.1	18.7 ± 16.5	0.18
PGNF	1 (4.2%)	1 (8.3%)	0.62
IPGD	11 (45.8%)	2 (16.7%)	0.07
Reoperation	3 (12.5%)	2 (16.7%)	0.74
Retrasplantation	2 (8.3%)	3 (25%)	0.17
Rejection	5 (20.8%)	0 (0%)	0.23
Artery complications	1 (4.2%)	1 (8.3%)	0.61
Biliary complications	3 (12.5%)	3 (25%)	0.34
Infections	3 (12.5%)	4 (33.3%)	0.28
Hospital re-stay	0 (0%)	1 (8.3%)	0.13
Mean patient survival			
1-year survival	95.8%	91.7%	NS
5--year survival	79.9%	80.2%	NS
Mean graft survival			
1-year survival	87.5%	83.3%	NS
3-year survival	87.5%	54.7%	NS
5--year survival	70%	27.3%	NS

Intensive care unit (ICU); primary graft non-function (PGNF); initial poor graft dysfunction (IPGD); NS: Non significant

In our series, the most frequent indication among recipients of livers from older donors was alcohol-abuse (66.7%) followed by liver cancer (25%). Infection with hepatitis C virus was a conditioning factor for candidate acceptance. Unlike other etiologies, there is evidence (11) that survival rates are significantly lower for patients with hepatitis C virus who receive a liver from an older donor. Therefore, livers from older donors should not be transplanted into HCV-positive recipients. This is supported by the results obtained in our study. 

The main problem is that disease progression may occur among liver cancer patients who belong to the group of patients who could benefit from the broadening of donor selection criteria (12).

In conclusion, acceptable patient survival rates are obtained with the transplantation of suboptimal organs from older donors, if appropriately selected (13,14). Nevertheless, livers from older donors should be prevailingly used for cancer patients –such as liver cancer patients– on the waiting list for transplantation.

## Conflict of interests

 The authors declare that they have no conflict of interest.
